# A New Analytical Formulation for the Electrophoretic Mobility of a Colloidal Sphere

**DOI:** 10.3390/e27040336

**Published:** 2025-03-24

**Authors:** Angela Casarella, Simon Gourdin-Bertin, Claire Chassagne

**Affiliations:** 1 Department of Architecture and Civil Engineering, Chalmers University of Technology, 412 96 Göteborg, Sweden; angela.casarella@chalmers.se; 2 Independent Researcher, F-75012 Paris, France; simon.n.gourdin@gmail.com; 3 Section of Environmental Fluid Mechanics, Department of Hydraulic Engineering, Delft University of Technology, 2628 CN Delft, The Netherlands

**Keywords:** electrophoresis, colloid, zeta potential, Hückel, Smoluchowski

## Abstract

A new analytical equation for the electrophoretic mobility of a colloidal sphere, homogeneously charged, is derived. This equation reduces to the well-known Henry’s formulation for low surface potentials. For high surface potentials, the equation is compared to the full numerical result. It is found that the equation performs well up to surface potentials of 50 mV. For larger surface potentials, the equation performs well for κa>10, where κ is the inverse of Debye’ s length and *a* the radius of the particle. Differences between analytical and numerical solutions for κa<10 are studied. The case of a particle with a constant surface charge is discussed. In that case, a very simple equation relates the surface charge of the particle to the electrophoretic mobility for κa>10.

## 1. Introduction

The motion of a charged colloidal particle in an electrolyte under the influence of an applied electric field is used routinely in many fields of science to study the changes in interfacial properties of colloids upon changes in solvent (changes in pH or changes in ionic strength, for example). In order to interpret the measured data, that is, the electrophoretic velocity defined as $\mu =U/E_{0}$ whereby *U* is the velocity of a colloidal particle subjected to an electric field $E_{0}$, one makes use of theories of which the simplest ones date from the work of Smoluchowski and Hückel [1]. A full numerical description of the electrophoretic mobility was proposed in 1978 by O’Brien and White [2]. Numerous analytical approximations have also been developed for different ranges of applicability: high and low ionic strength, low surface charge, and particles coated by polyelectrolytes, for example [1]. Recently, there was regained interest in the physical aspects of electrophoresis. Jayaraman et al. pointed out the unusual fluid dynamics around a charged colloidal particle and provided an overview of different theories [3]. One of the authors of the article even translated the original PhD thesis of Overbeek from Dutch to English to help the modern reader bring this original work into context (see web link in [3]).

In the present article, we would like to make the connection between these theories and the approach taken in the work of Chassagne and Bedeaux [4] to study the polarization of a double layer around a charged colloidal particle. In particular, we would like to make the link between electrophoretic mobility and dipole coefficient β, where β is related to the dipole moment P (see Equation (41)) generated from the application of the electric field $E_{0}$. The main finding of the present article is Equation (75), where μ is shown to be a linear function of β. This equation is an improved version of a similar equation presented in [4]. In the present article, we provide a mathematical derivation for Equation (75) (which was not conducted in [4]) and discuss the validity of the three main hypotheses formulated to obtain the equation. We hereby extend the analysis conducted by Jayaraman et al. [3] and describe the behavior of the electric potential and electrochemical potentials for specific cases, such as low and high ionic strengths and low and high surface electric charges. The particular case of a particle with a constant surface charge is discussed, which is a case that is often encountered in practice.

## 2. Definitions

In this section, we define the required variables and set up the relations that will be solved in the following sections. A charged spherical and dielectric particle of radius *a*, homogeneously charged, is subjected to an oscillating electric field $E_{0}=E_{0}\exp\left(i\omega t\right)e_{z}$ of radial frequency ω (rad/s). In this article, we focus on the case ω=0. More general equations can be found in [4]. The vector $e_{z}$ represents a unity vector along the z-axis, where (x,y,z) designs the usual Cartesian coordinates and $\left(e_{r},e_{\theta },e_{\varphi }\right)$ are the unit vectors in spherical coordinates. We place ourselves in the frame of reference of the particle, and the origin of the coordinate system is the center of the considered particle, whereby the polar axis (θ=0) is set parallel to $E_{0}$ and hence

(1) $$E_{0}=E_{0}e_{z}=E_{0}\cos\theta e_{r}-E_{0}\sin\theta e_{\theta }$$

The particle is immersed in an electrolyte solution composed of one type of positively charged ions and one type of negatively charged ions.

The main assumptions used in the derivation are as follows.

1. The Reynolds number is small so that the inertial terms in the Navier–Stokes equations can be ignored.
2. The fluid is incompressible.
3. The applied electric field is weak compared to the local electric field around the particle so that higher-order terms in $E_{0}$ can be neglected.

In the present article, we assume that

(2) $$\Psi_{eq}\left(a\right)=\zeta$$

where $\Psi_{eq}$ is the equilibrium potential in the absence of applied electric field. The zeta potential ζ is defined as the electric potential at the surface of shear. By assuming Equation (2) to be valid, we imply that the surface of shear that will be created when the particle is in motion is located on the surface of the particle. We will also assume that there is no Stern layer. The Stern layer is defined as a very small region of space at the interface between the particle and the electrolyte where solvent and ionic properties could deviate from their bulk values. This assumption implies that the equations that will be given in the next sections are valid between r=a (the particle’s surface) and infinity. The general relations for the electric potential Ψ and the ionic densities $n_{i,tot}$ (i=+,−) are given by

(3) $$\begin{array}{ccc} \Psi \left(r,\theta \right) & = & \Psi_{eq}\left(r\right)+\delta \Psi \left(r,\theta \right)\\ n_{+,tot}\left(r,\theta \right) & = & n_{+,eq}\left(r\right)+\delta n_{+}\left(r,\theta \right)\\ n_{-,tot}\left(r,\theta \right) & = & n_{-,eq}\left(r\right)+\delta n_{-}\left(r,\theta \right) \end{array}$$

where $n_{i,eq}$ represents the ionic density of positively/negatively charged ions (number of ions/m^3^) of valence $z_{i}$ and stoichiometric coefficient $\nu_{i}$ in absence of applied electric field. Because of electroneutrality,

(4) $$\sum_{i}\nu_{i}z_{i}=0$$

The functions δΨ, $\delta n_{+}$, and $\delta n_{-}$ represent the contributions due to the presence of the applied electric field.

Due to axial symmetry, one can define

(5) $$\begin{array}{ccc} \delta \Psi \left(r,\theta \right) & = & \left(\psi \left(r\right)-r\right)E_{0}\cdot e_{r}\\ \delta n_{+}\left(r,\theta \right) & = & n_{+}\left(r\right)E_{0}\cdot e_{r}\\ \delta n_{-}\left(r,\theta \right) & = & n_{-}\left(r\right)E_{0}\cdot e_{r} \end{array}$$

It follows that the electric field resulting from the application of $E_{0}$ is

(6) $$\begin{array}{ccc} \delta E_{r} & = & -\left(\frac{d\psi }{dr}-1\right)E_{0}\cos\left(\theta \right)\\ \delta E_{\theta } & = & \left(\frac{\psi }{r}-1\right)E_{0}\sin\left(\theta \right) \end{array}$$

### 2.1. The Double Layer

The so-called double layer surrounding the colloidal particle is composed of (a) a first layer composed of the surface charges grafted onto the colloidal particle and (b) a second layer (the diffuse layer) dominated by an excess of counter-ions (and a depletion of co-ions) that are electrically interacting with the surface charges. As done by many authors, the term “double layer” will be used when “diffuse layer” is implied. Beyond the double layer, in the absence of an applied electric field, the concentrations of counter and co-ions are such that electroneutrality (see Equation (4)), is respected.

The thickness of the diffuse layer is given by the Debye length, which is defined by

(7) $$\kappa^{-1}=\sqrt{\frac{\varepsilon_{0}\varepsilon_{1}kT}{e^{2}n_{\infty }\sum z_{i}^{2}\nu_{i}}}$$

with

(8) $$n_{\infty }=\mathrm{Cs}\times N_{A}$$

where Cs is the neutral salt concentration in mM (10^−3^ mol/L), N_A_ is Avogadro’s number, $\varepsilon_{0}$ is the permittivity of vacuum, and $\varepsilon_{1}$ is the relative permittivity of the solvent (which will be water in the present article).

Beyond the double layer (in the bulk), we have

$$n_{i,eq}\left(r\gg a+\kappa^{-1}\right)=\nu_{i}n_{\infty }$$

### 2.2. The Electrochemical Potential

The electrochemical potential ${\tilde{\mu }}_{i}$ is defined by

(9) $${\tilde{\mu }}_{i}=z_{i}e\Psi +\mu_{i}$$

The chemical potential $\mu_{i}$ is defined by

(10) $$\mu_{i}=\mu_{i}^{0}+kT\ln\left(\frac{n_{i,tot}}{n_{0}}\right)$$

where $\mu_{i}^{0}$ and $n_{0}$ are reference values, *k* is the Boltzmann constant, and *T* is the temperature. Note that it is the fact that the electrochemical potentials are constant in the absence of applied electric field, i.e.,

(11) $${\tilde{\mu }}_{i,eq}\left(r\right)={\tilde{\mu }}_{i,eq}\left(\infty \right)$$

that leads to the Boltzmann distribution:

(12) $$n_{i,eq}=\nu_{i}n_{\infty }\exp\left(\frac{-z_{i}e\Psi_{eq}}{kT}\right)$$

The electrochemical potential can also be written

(13) $$\begin{array}{ccc} {\tilde{\mu }}_{i} & = & {\tilde{\mu }}_{i.eq}+\delta {\tilde{\mu }}_{i}\\ & = & z_{i}e\left(\Psi_{eq}+\delta \Psi \right)+\mu_{i}^{0}+kT\ln\left(\frac{n_{i,eq}+\delta n_{i}}{n_{0}}\right) \end{array}$$

One can show that

(14) $$\delta {\tilde{\mu }}_{i}=z_{i}e\delta \Psi +kT\frac{\delta n_{i}}{n_{i,eq}}$$

Because of symmetry, we introduce the variable $\phi_{i}$ such that

(15) $$\begin{array}{ccc} \delta {\tilde{\mu }}_{i}\left(r\right) & = & -z_{i}e\left[\phi_{i}\left(r\right)+r\right]E_{0}\cos\theta \\ & = & z_{i}e\left[\psi \left(r\right)-r\right]E_{0}\cos\theta +kT\frac{n_{i}\left(r\right)}{n_{i,eq}\left(r\right)}E_{0}\cos\theta \end{array}$$

This implies that

(16) $$n_{i}=\frac{-z_{i}en_{i,eq}}{kT}\left(\psi +\phi_{i}\right)$$

### 2.3. The Ionic Flux

The ionic flux $J_{i}$ is given by

(17) $$J_{i}=n_{i,eq}u-\frac{D_{i}n_{i}}{kT}\nabla {\tilde{\mu }}_{i}$$

where u is the fluid velocity in the reference frame of the particle, and $D_{i}$ (m^2^/s) is the ionic diffusion coefficient. The ionic fluxes at equilibrium are defined by

(18) $$J_{i,eq}=\frac{-D_{i}n_{i,eq}}{kT}\nabla {\tilde{\mu }}_{i,eq}=0$$

The fact that the electrochemical potentials are constant in the absence of an applied electric field is correlated to $J_{i,eq}=0$, which leads to the Boltzmann distribution, Equation (12). The fluxes due to the application of the electric field are, to first order, given by

(19) $$\delta J_{i}=n_{i,eq}u-\frac{D_{i}n_{i,eq}}{kT}\nabla \delta {\tilde{\mu }}_{i}$$

### 2.4. The Velocity

The condition ∇·u=0 (which arises from the fact that the fluid is incompressible) is used to express u(r,θ) as function of a new function h(r) such that [1]

(20) $$\begin{array}{ccc} u & = & \frac{-2}{r}hE\cos\theta e_{r}+\frac{1}{r}\frac{d}{dr}\left[rh\right]E_{0}\sin\theta e_{\theta } \end{array}$$

The calculations are conducted in the reference frame of the particle, which implies that

$$\begin{array}{ccc} u\left(r=a\right) & = & 0\;\;\;\;(\mathrm{no}\;\mathrm{slip})\\ u\left(r\to \infty \right) & = & -\mu E_{0}\;\;\;\;(\mathrm{far}\;\mathrm{away},\;\mathrm{the}\;\mathrm{fluid}\;\mathrm{moves}\;\mathrm{at}\;\mathrm{minus}\;\mathrm{the}\;\mathrm{velocity}\;\mathrm{of}\;\mathrm{the}\;\mathrm{particle}) \end{array}$$

where

(21) $$\mu =\frac{U}{E_{0}}$$

is called electrophoretic mobility (note that its symbol μ should not be confused with $\mu_{i}$, which is used for the ionic chemical potential).

### 2.5. Variable Definitions

For convenience, we use the following dimensionless variables:

$$\begin{array}{lll} \mathrm{no}\;\dim. & & \mathrm{with}\;\dim.\\ {\hat{\Psi }}_{eq} & = & e\Psi_{eq}/\left(kT\right)\\ \hat{\psi } & = & \kappa \psi \\ \hat{\phi_{i}} & = & \kappa \phi_{i}\\ x & = & \kappa r\\ \hat{h} & = & h\left(e\eta \kappa \right)/\left(\varepsilon_{0}\varepsilon_{1}kT\right)\\ \hat{M} & = & M\left(e\eta \right)/\left(\kappa^{3}\varepsilon_{0}\varepsilon_{1}kT\right)\\ \hat{\mu } & = & \mu \left(e\eta \right)/\left(\varepsilon_{0}\varepsilon_{1}kT\right)\\ \hat{D_{i}} & = & D_{i}\left(e^{2}\eta \right)/\left(\varepsilon_{0}\varepsilon_{1}{\left(kT\right)}^{2}\right) \end{array}$$

## 3. The Poisson–Boltzmann Equation

The Poisson–Boltzmann equation is given by

(22) $$\nabla^{2}\Psi =\frac{-1}{\varepsilon_{0}\varepsilon_{1}}\sum ez_{i}n_{i,tot}$$

For the equilibrium part, using Equation (12) and dimensionless variables, one obtains

(23) $$\begin{array}{ccc} \frac{d^{2}{\hat{\Psi }}_{eq}}{dx^{2}}+\frac{2}{x}\frac{d{\hat{\Psi }}_{eq}}{dx} & = & \frac{-1}{\sum z_{i}^{2}\nu_{i}}\sum z_{i}\nu_{i}\exp\left(-z_{i}{\hat{\Psi }}_{eq}\right)\\ & = & \sinh({\hat{\Psi }}_{eq})\;\;\mathrm{for}\;a\;1–1\;\mathrm{electrolyte} \end{array}$$

The Poisson–Boltzmann for the potential arising from the applied electric field is obtained by using Equations (12) and (16)

(24) $$\begin{array}{ccc} \hat{L}\hat{\psi } & = & \frac{d^{2}\hat{\psi }}{dx^{2}}+\frac{2}{x}\frac{d\hat{\psi }}{dx}-\frac{2\hat{\psi }}{x^{2}}=\frac{1}{\sum z_{i}^{2}\nu_{i}}\sum z_{i}^{2}\nu_{i}\exp\left(-z_{i}{\hat{\Psi }}_{eq}\right)\left[\hat{\psi }+\hat{\phi_{i}}\right] \end{array}$$

(25) $$\begin{array}{ccc} & = & \cosh\left({\hat{\Psi }}_{eq}\right)\left[\hat{\psi }+\hat{\phi_{i}}\right]\;\;\mathrm{for}\;a\;1–1\;\mathrm{electrolyte} \end{array}$$

The operator *L* (with $\hat{L}=L/\kappa^{2}$) is defined on an arbitrary function g(r) by

(26) $$Lg\left(r\right)=\frac{1}{r}\frac{d^{2}}{dr^{2}}\left(rg\left(r\right)\right)-\frac{2g\left(r\right)}{r^{2}}$$

### 3.1. Boundary Conditions at r=a

The boundary condition for the equilibrium electric potential ${\hat{\Psi }}_{eq}$ is given by

(27) $${\hat{\Psi }}_{eq}\left(x=\kappa a\right)={\hat{\Psi }}_{0}$$

where ${\hat{\Psi }}_{0}=\hat{\zeta }$ is the zeta potential in case there is no Stern layer and that the slip plane is located at the surface of the particle.

The boundary condition for the equilibrium electric potential $\Psi_{eq}$ is also given by, following Gauss,

(28) $$\varepsilon_{0}\varepsilon_{2}{\left(\frac{d\Psi_{2,eq}}{dr}\right)}_{r=a}-\varepsilon_{0}\varepsilon_{1}{\left(\frac{d\Psi_{eq}}{dr}\right)}_{r=a}=\sigma_{s}$$

where $\sigma_{s}$ (C/m^2^) is the surface charge density, $\varepsilon_{2}$ is the relative permittivity of the particle, and $\Psi_{eq,2}$ is the electric potential inside the particle. This potential satisfies the Laplace’s equation ($\nabla^{2}\Psi_{2,eq}=0$). The solution of the Laplace’s equation is given in terms of Legendre polynomials (see p. 350 in [5]), and, because $\Psi_{2,eq}$ cannot be singular in r=0 nor be θ-dependent, it follows that $\Psi_{2,eq}$ should be a constant. From the continuity of electric potentials, i.e.,

(29) $$\Psi_{2,eq}\left(r=a^{-}\right)=\Psi_{eq}\left(r=a^{+}\right)$$

it follows that

(30) $${\hat{\Psi }}_{2,eq}\left(x\right)={\hat{\Psi }}_{0}$$

The surface charge density $\sigma_{s}$ can usually be assessed by titration. For most colloids, one usually has $\varepsilon_{2}\ll \varepsilon_{1}$, implying that Equation (28) reduces to

(31) $${\left(\frac{d\Psi_{eq}}{dr}\right)}_{r=a}=\frac{-\sigma_{s}}{\varepsilon_{0}\varepsilon_{1}}$$

For specific suspensions such as charged sulfate latex colloidal spheres at a given pH, the surface charge density is not expected to vary when the electrolyte concentration is varied [6]. Equation (31) can be used to estimate the surface charge density $\sigma_{s}$, while the potential ${\hat{\Psi }}_{0}$ can be estimated from electrophoretic mobility data [6]. Discrepancies between measured data and prediction are associated with the thin layer of fluid at the particle/electrolyte interface (Stern layer). In the Stern layer, both the ionic mobility and the dielectric permittivity differ from their bulk values [7], and, for highly charged particles, the use of Equation (12) can lead to unrealistically high ionic concentrations close to the particle’s surface, as a simple estimation can show. A Stern layer parameter can then be introduced in the equations to account for the deviation from ideality. We will not consider Stern layers in the present article.

The boundary conditions for δΨ are given by

(32) $$\begin{array}{ccc} \varepsilon_{0}\varepsilon_{2}{\left(\frac{d\left(\delta \Psi_{2}\right)}{dr}\right)}_{r=a} & = & \varepsilon_{0}\varepsilon_{1}{\left(\frac{d\left(\delta \Psi \right)}{dr}\right)}_{r=a}\\ \delta \Psi_{2}\left(a\right) & = & \delta \Psi (a) \end{array}$$

$\delta \Psi_{2}$ is the potential inside the dielectric sphere due to applied electric field and is solution of Laplace’s equation ($\Delta \delta \Psi_{2}=0$), which yields

(33) $$\delta \Psi_{2}=-E_{2}r\cos\theta$$

Eliminating $E_{2}$ in the equations leaves

(34) $${\left(\frac{d\left(\delta \Psi \right)}{dr}\right)}_{r=a}=\frac{\varepsilon_{2}}{\varepsilon_{1}}\frac{\delta \Psi (a)}{a}$$

which yields

(35) $${\left(\frac{d\hat{\psi }}{dx}\right)}_{x=\kappa a}-1=\frac{\varepsilon_{2}}{\varepsilon_{1}}\frac{\hat{\psi }\left(\kappa a\right)-\kappa a}{\kappa a}$$

The magnitude of the electric field inside the colloidal particle is given by

(36) $$E_{2}=\left(1-\frac{\psi \left(a\right)}{a}\right)E_{0}$$

### 3.2. Boundary Conditions Far Away

Far away from the particle, one finds, for any electrolyte,

$$\frac{d^{2}{\hat{\Psi }}_{eq}}{dx^{2}}+\frac{2}{x}\frac{d{\hat{\Psi }}_{eq}}{dx}=\frac{-1}{\sum z_{i}^{2}\nu_{i}}\sum z_{i}\nu_{i}\left(1-z_{i}{\hat{\Psi }}_{eq}\right)={\hat{\Psi }}_{eq}\;\mathrm{for}\;x\gg \kappa a+1$$

yielding

(37) $${\hat{\Psi }}_{eq}^{+}\left(x\right)={\hat{\Psi }}_{ap}\frac{\kappa a}{x}\exp\left(\kappa a-x\right)$$

where ${\hat{\Psi }}_{ap}$ is an apparent surface potential as it would be the surface potential if the relation Equation (37) would hold until x=κa (i.e., r=a). Equation (37) holds for any *x* for low surface potentials (${\hat{\Psi }}_{0}={\hat{\Psi }}_{eq}^{+}\left(\kappa a\right)$) as then the approximation $\exp\left(-z_{i}{\hat{\Psi }}_{eq}\right)=1-z_{i}{\hat{\Psi }}_{eq}$ is valid. It follows that, for low surface potentials ${\hat{\Psi }}_{0}$,

(38) $$\begin{array}{ccc} {\hat{\Psi }}_{eq}\left(x\right) & = & {\hat{\Psi }}_{0}\frac{\kappa a}{x}\exp\left(\kappa a-x\right)\;\mathrm{for}\;\mathrm{any}\;x\\ {\hat{\Psi }}_{ap} & = & {\hat{\Psi }}_{0}\;\mathrm{for}\;{\hat{\Psi }}_{0}\ll 1 \end{array}$$

Assymptotically, for x→∞, one defines

(39) $${\hat{\Psi }}_{eq}^{\infty }=0$$

The assymptotic value of $\psi^{+}$ is given by (see Appendix A)

(40) $${\hat{\psi }}^{+}=\frac{{\left(\kappa a\right)}^{3}\beta }{x^{2}}$$

where β is the dipole coefficient, related to the dipole moment

(41) $$P=4\pi \varepsilon_{0}\varepsilon_{1}a^{3}\beta E_{0}$$

generated from the application of the electric field. The dipolar nature of the system composed of the charged colloidal particle and its double layer is illustrated in Appendix D, where the results of COMSOL calculations are plotted. The assymptotic form of the electric potential is given by

(42) $$\delta \psi^{+}\left(r\right)=\left(-r+\frac{a^{3}\beta }{r^{2}}\right)E_{0}\cos\theta$$

The approximate expressions for β are given in Appendix B. More general relations are given in [8].

## 4. The Conservation of Mass Law (Nernst–Planck)

The law of conservation of mass, for the DC case considered here (which implies that $\partial n_{i}/\partial t=0$), can be expressed as

(43) $$\nabla \cdot J_{i}=0$$

To first order, the ionic flux becomes

(44) $$\begin{array}{ccc} J_{i} & = & n_{i,eq}u-D_{i}n_{i,eq}\nabla \delta \tilde{\mu_{i}}\\ \nabla \delta \tilde{\mu_{i}} & = & \nabla \left(\frac{z_{i}e}{kT}\delta \Psi +\frac{\delta n_{i}}{n_{i,eq}}\right) \end{array}$$

One can show that

(45) $$\begin{array}{ccc} & & \nabla .\left[n_{i,eq}\nabla \delta \tilde{\mu_{i}}\right]=\nabla n_{i,eq}\cdot \nabla \delta \tilde{\mu_{i}}+n_{i,eq}\nabla^{2}\delta \tilde{\mu_{i}}\\ & = & \\ & & z_{i}en_{i,eq}\left(\frac{z_{i}e}{kT}\frac{d\Psi_{eq}}{dr}\left(\frac{\partial \phi_{i}}{\partial r}+1\right)-\left[\frac{\partial^{2}\phi_{i}}{\partial r^{2}}+\frac{2}{r}\frac{\partial \phi_{i}}{\partial r}-\frac{2}{r^{2}}\phi_{i}\right]\right)E_{0}\cos\theta \end{array}$$

This implies that the law of mass conservation can be written

(46) $$\hat{L}\hat{\phi_{i}}=\frac{\partial^{2}\hat{\phi_{i}}}{\partial x^{2}}+\frac{2}{x}\frac{\partial \hat{\phi_{i}}}{\partial x}-\frac{2}{x^{2}}\hat{\phi_{i}}=z_{i}\frac{d\Psi_{eq}}{dx}\left[\frac{d\hat{\phi_{i}}}{dx}-\frac{1}{z_{i}\hat{D_{i}}}\frac{2\hat{h}}{x}+1\right]$$

### 4.1. Boundary Conditions at r=a

There is no ionic flux possible perpendicular to the surface of the particle; hence,

(47) $$\begin{array}{ccc} {\left(J_{i}\cdot e_{r}\right)}_{r=a} & = & 0\\ {\left(\nabla {\tilde{\mu }}_{i}\cdot e_{r}\right)}_{r=a} & = & 0 \end{array}$$

where we have used the no-slip condition

(48) $$u\left(r=a\right)=0$$

We obtain

(49) $${\left(\frac{d\hat{\phi_{i}}}{dx}\right)}_{x=\kappa a}=-1$$

### 4.2. Boundary Conditions Far Away

In Appendix A, it was found that, for x≫κa+1,

(50) $${\hat{\phi }}_{+}^{+}={\hat{\phi }}_{-}^{+}=-\frac{{\left(\kappa a\right)}^{3}\beta }{x^{2}}$$

## 5. Navier–Stokes

The Navier–Stokes equation provides the last fundamental equation required to solve the problem:

(51) $$\eta \nabla^{2}u-\nabla P=\sum ez_{i}n_{i}\nabla \Psi$$

where *P* is the pressure. We used the fact that the Reynolds number Re $=\left|\rho_{m}\left(u\cdot \nabla \right)u\right|/\left|\eta \nabla^{2}u\right|$ and also $\left|\rho_{m}\partial u/\partial t\right|/\left|\eta \nabla^{2}u\right|$ are very small compared to one. To first order, the Navier–Stokes reduces to

(52) $$\eta \nabla^{2}u-\nabla P=\sum ez_{i}\left[\delta n_{i}\nabla \Psi_{eq}+n_{i,eq}\nabla \delta \Psi \right]$$

In order to get rid of the pressure term P, we take the curl of the Navier–Stokes equation:

(53) $$\eta \nabla^{2}\left(\nabla \times u\right)=\sum ez_{i}\nabla \times \left[n_{i}\nabla \Psi \right]$$

For a discussion about the pressure and the peculiar form of the velocity u, we refer to [3,9]. Equation (53) can be written

(54) $$\eta \nabla^{2}\left(\nabla \times u\right)=\sum ez_{i}\left[\nabla \delta n_{i}\times \nabla \Psi_{eq}+\nabla n_{i,eq}\times \nabla \delta \Psi \right]$$

One can show that

(55) $$\begin{array}{ccc} & & \nabla \delta n_{i}\times \nabla \Psi_{eq}+\nabla n_{i,eq}\times \nabla \delta \Psi \\ & = & \frac{-z_{i}en_{i,eq}}{kT}\left(\frac{\phi_{i}}{r}+1\right)\frac{d\Psi_{eq}}{dr}\sin\theta E_{0}e_{\phi } \end{array}$$

and that

(56) $$\nabla^{2}\left(\nabla \times u\right)=\left[\frac{\partial^{4}h}{\partial r^{4}}+\frac{4}{r}\frac{\partial^{3}h}{\partial r^{3}}-\frac{4}{r^{2}}\frac{\partial^{2}h}{\partial r^{2}}\right]\sin\theta E_{0}e_{\phi }$$

This implies that the Navier–Stokes reduces to

(57) $$\hat{L}\hat{L}\hat{h}=\left[\frac{\partial^{4}\hat{h}}{\partial x^{4}}+\frac{4}{x}\frac{\partial^{3}\hat{h}}{\partial x^{3}}-\frac{4}{x^{2}}\frac{\partial^{2}\hat{h}}{\partial x^{2}}\right]=\frac{d{\hat{\Psi }}_{eq}}{dx}\frac{-1}{\sum z_{i}^{2}\nu_{i}}\sum z_{i}^{2}\nu_{i}\exp\left(-z_{i}{\hat{\Psi }}_{eq}\right)\left(\frac{\hat{\phi_{i}}}{x}+1\right)$$

Note that, in the case that the colloidal sphere is uncharged but placed in a flow field such that $u\left(r=a\right)=0$ and $u\left(r\to \infty \right)=-U$, the hydrodynamics are described by the equation

LLh=0

The solution of this equation is

(58) $$h\left(r\right)=\frac{U}{2}\left(r-\frac{3}{2}a+\frac{a^{3}}{2r^{2}}\right)$$

yielding the equation for a Stokes flow around a sphere:

(59) $$u\left(r\right)=-U\left(1-\frac{3}{2r}a+\frac{a^{3}}{2r^{3}}\right)\cos\theta e_{r}+U\left(1-\frac{3}{4r}a+\frac{a^{3}}{4r^{3}}\right)\sin\theta e_{\theta }$$

### 5.1. Boundary Conditions at r=a

From the no-slip condition,

(60) $$u\left(r=a\right)=0$$

we obtain

(61) $$\begin{array}{ccc} \hat{h}\left(\kappa a\right) & = & 0 \end{array}$$

(62) $$\begin{array}{ccc} {\left(\frac{d\hat{h}}{dr}\right)}_{x=\kappa a} & = & 0 \end{array}$$

### 5.2. Boundary Conditions Far Away

Far away from the particle,

(63) $$u\left(r\to \infty \right)=-\mu E_{0}$$

yielding

(64) $$\begin{array}{ccc} \frac{2}{x}\hat{h} & = & \hat{\mu }\;\mathrm{for}\;x\to \infty \end{array}$$

(65) $$\begin{array}{ccc} \frac{1}{x}\frac{d}{dx}\left[x\hat{h}\right] & = & \hat{\mu }\;\mathrm{for}\;x\to \infty \end{array}$$

## 6. Analytical Solutions

### 6.1. Full Solution as Function of Integrals

Equation (57) can be solved analytically using boundary conditions Equations (61), (62), (64) and (65). The solution of this fourth-order linear differential equation can be obtained using the method of variation of parameters and the Wronskian of the general solutions (for the method, see [10], p. 331) to yield

(66) $$\begin{array}{ccc} \hat{h}\left(x\right) & = & \int_{\kappa a}^{\infty }\left[-\frac{x^{3}}{30}-\frac{{\left(\kappa a\right)}^{5}}{20x^{2}}+\frac{{\left(\kappa a\right)}^{3}}{12}+y^{2}\left(\frac{x}{6}+\frac{{\left(\kappa a\right)}^{3}}{12x^{2}}-\frac{\left(\kappa a\right)}{4}\right)\right]\hat{M}\left(y\right)dy\\ & & +\frac{\hat{\mu }}{2}\left(x-\frac{3}{2}\kappa a+\frac{{\left(\kappa a\right)}^{3}}{2x^{2}}\right)+\int_{\kappa a}^{x}\left(\frac{x^{3}}{30}-\frac{xy^{2}}{6}+\frac{y^{3}}{6}-\frac{y^{5}}{30x^{2}}\right)\hat{M}\left(y\right)dy \end{array}$$

with

(67) $$\hat{\mu }=\frac{{\left(\kappa a\right)}^{2}}{9}\int_{\kappa a}^{\infty }\left(1-3\frac{x^{2}}{{\left(\kappa a\right)}^{2}}+2\frac{x^{3}}{{\left(\kappa a\right)}^{3}}\right)\hat{M}\left(x\right)dx$$

and

(68) $$\begin{array}{ccc} \hat{M}\left(x\right) & = & \frac{d{\hat{\Psi }}_{eq}}{dx}\frac{-1}{\sum z_{i}^{2}\nu_{i}}\sum z_{i}^{2}\nu_{i}\exp\left(-z_{i}{\hat{\Psi }}_{eq}\right)\left(\frac{\hat{\phi_{i}}}{x}+1\right)\\ & = & \frac{d{\hat{\Psi }}_{eq}}{dx}\frac{-1}{2}\sum \exp\left(-z_{i}{\hat{\Psi }}_{eq}\right)\left(\frac{\hat{\phi_{i}}}{x}+1\right)\;\mathrm{for}\;a\;1–1\;\mathrm{electrolyte} \end{array}$$

The term that multiplies the term $\hat{\mu }/2$ is the same as the one found for the Stokes flow in Equation (58). One can verify that combining Equations (66) and (67) yields the solutions presented by both Ohshima [11] and Jayaraman et al. [3], which they write (inserting Equation (67) in Equation (66))

(69) $$\begin{array}{ccc} \hat{h}\left(x\right) & = & -\left(\frac{x^{3}}{30}+\frac{{\left(\kappa a\right)}^{5}}{45x^{2}}-\frac{{\left(\kappa a\right)}^{2}x}{18}\right)\int_{\kappa a}^{\infty }\hat{M}\left(y\right)dy+\int_{\kappa a}^{x}\left(\frac{x^{3}}{30}-\frac{xy^{2}}{6}+\frac{y^{3}}{6}-\frac{y^{5}}{30x^{2}}\right)\hat{M}\left(y\right)dy\\ & & +\int_{\kappa a}^{\infty }\left(\frac{x}{9\kappa a}-\frac{1}{6}+\frac{{\left(\kappa a\right)}^{2}}{18x^{2}}\right)y^{3}\hat{M}\left(y\right)dy \end{array}$$

### 6.2. Approximated Analytical Solution

As a first approximation, we will use the relation Equation (50) assuming that $\hat{\phi_{i}}$ can be approximated by $\hat{\phi_{i}^{+}}$ inside the double layer. It follows that

(70) $$\begin{array}{ccc} \hat{M}\left(x\right) & ∼ & \left(1-\frac{{\left(\kappa a\right)}^{3}}{x^{3}}\beta \right)\frac{1}{\sum z_{i}^{2}\nu_{i}}\frac{d}{dx}\left[\sum z_{i}\nu_{i}\exp\left(-z_{i}{\hat{\Psi }}_{eq}\right)\right]\\ \hat{M}\left(x\right) & ∼ & \left(1-\frac{{\left(\kappa a\right)}^{3}}{x^{3}}\beta \right)\frac{d{\hat{\Psi }}_{eq}}{dx}\left[\cosh({\hat{\Psi }}_{eq})\right]\;\mathrm{for}\;a\;1–1\;\mathrm{electrolyte} \end{array}$$

We will now follow the procedure adopted by Ohshima (see p. 83 in [1]), who made the observation that the function

(71) $$\left(\frac{\hat{\phi_{i}}}{x}+1\right)∼\left(1-\frac{{\left(\kappa a\right)}^{3}}{x^{3}}\beta \right)$$

is varying very slowly compared to the other functions and can hence be set outside the integral. We note that this assumption is equivalent to assume that there is a local equilibrium and that the electrochemical potentials (we recall that $\delta {\tilde{\mu }}_{i}=-z_{i}e\left[\phi_{i}\left(r\right)+r\right])$ are slowly varying through the double layer. This assumption was already tested in [4], where it was found that it did not hold for low κa.

Using the assumption, we find

(72) $$\hat{\mu }=\frac{{\left(\kappa a\right)}^{2}}{9}\left(1-\frac{{\left(\kappa a\right)}^{3}}{x_{1}^{3}}\beta \right)\int_{\kappa a}^{\infty }\left(1-3\frac{x^{2}}{{\left(\kappa a\right)}^{2}}+2\frac{x^{3}}{{\left(\kappa a\right)}^{3}}\right)\frac{1}{\sum z_{i}^{2}\nu_{i}}\frac{d}{dx}\left[\sum z_{i}\nu_{i}\exp\left(-z_{i}{\hat{\Psi }}_{eq}\right)\right]dx$$

where $x_{1}$ is the position where the function to be integrated is maximum. Ohshima has a less general expression as he studies the case where ${\hat{\Psi }}_{0}$ is small. In Ohshima’s case, β=−1/2, which corresponds to the case ${\hat{\Psi }}_{0}\ll 1$ [4].

Integrating by parts, it is found that

(73) $$\hat{\mu }=\frac{2}{3}\left(1-\frac{{\left(\kappa a\right)}^{3}}{x_{1}^{3}}\beta \right)\int_{\kappa a}^{\infty }\left(\left(1-\frac{x}{\left(\kappa a\right)}\right)\right)\frac{x}{\sum z_{i}^{2}\nu_{i}}\left[\sum z_{i}\nu_{i}\exp\left(-z_{i}{\hat{\Psi }}_{eq}\right)\right]dx$$

We now make the approximation that ${\hat{\Psi }}_{eq}\left(x\right)$ can be approximated by ${\hat{\Psi }}_{eq}^{+}\left(x\right)$ in the double layer (which also implies that we assume that ${\hat{\Psi }}_{app}∼{\hat{\Psi }}_{0})$ and use Equation (37) to estimate ${\hat{\Psi }}_{eq}$. This yields

(74) $$\hat{\mu }=\frac{-2\kappa a}{3}{\hat{\Psi }}_{0}\left(1-\frac{{\left(\kappa a\right)}^{3}}{x_{1}^{3}}\beta \right)\left[1-\frac{1}{\kappa a}\int_{\kappa a}^{\infty }x\exp\left(\kappa a-x\right)dx\right]$$

Integrating by parts, one obtains

(75) $$\hat{\mu }=\frac{2}{3}{\hat{\Psi }}_{0}\left(1-\frac{{\left(\kappa a\right)}^{3}}{x_{1}^{3}}\beta \right)$$

We have found, inspired by Ohshima [1] and trial and error, that a good estimation of $x_{1}$ for a large range of κa and ${\hat{\Psi }}_{0}$ is given by (see Appendix C for a discussion on $x_{1}$)

(76) $$x_{1}=\kappa a+\frac{2.5}{1+2\exp\left(-\kappa a\right)\exp\left(-{\hat{\Psi }}_{0}\right)}$$

In the following section, the comparison between Equation (75) and the numerical solution will be discussed. In particular, the following three hypotheses, formulated above, should be studied:

**Hypothesis 1.**  *$\hat{\phi_{i}}$ can be approximated by $\hat{\phi_{i}^{+}}$ (given by Equation (50)) inside the double layer.*

**Hypothesis 2.**  *$\left(\hat{\phi_{i}}/x+1\right)$ varies very slowly compared to the other functions in Equation (70).*

**Hypothesis 3.**  *${\hat{\Psi }}_{eq}$ can be approximated by ${\hat{\Psi }}_{eq}^{+}$ (given by Equation (37)) in the double layer.*

It can already be anticipated that these conditions hold for low ${\hat{\Psi }}_{0}$ (for which ${\hat{\Psi }}_{app}={\hat{\Psi }}_{0}$) for all κa as this has already been demonstrated by Ohshima [1]. Note that Ohshima uses

(77) $$x_{1}=\kappa a+\frac{2.5}{1+2\exp\left(-\kappa a\right)}$$

which reduces to Equation (76) for low ${\hat{\Psi }}_{0}.$

## 7. Comparison Between the Use of Equations (76) and (77)

The numerical results presented in this section (symbols) are obtained using a FORTRAN code, which solves the appropriate set of electrokinetic equations using the method from [12], which is an improvement on the Nordsieck method used previously in [4]. In particular, this numerical method enables studying the range of small κa, which was not possible using the Nordsieck method. Additional numerical calculations were performed using the Finite Element software COMSOL Multiphysics v. 6.3 ([13]). Some of the spatial representations of the analyzed quantities for selected values of κa are given in Appendix D. In all cases, the calculations performed using COMSOL match those obtained using FORTRAN. We emphasize that one of the hypotheses of the article is that we do not consider any Stern layer, and that the slip plane is located on the surface of the particle, implying that ${\hat{\Psi }}_{0}=\hat{\zeta }$ (the surface electric potential is the zeta potential).

In [4], it was found that Equation (75) using Equation (77) did not perform well at low applied electric field frequencies (see Figure 4A in [4]) but was a good match for high electric field frequencies (see Figure 4C in [4]). The mobility and surface electric potential are linked by a function that we define as Henry’s function $f_{\mathrm{Henry}}$:

(78) $$\hat{\mu }=f_{\mathrm{Henry}}{\hat{\Psi }}_{0}$$

**Figure 1:**
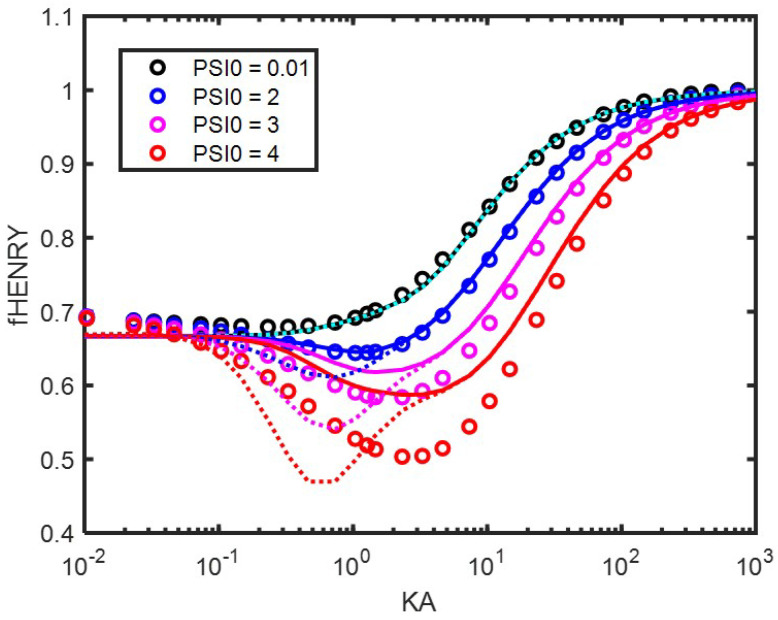
Henry’s function $f_{\mathrm{Henry}}$ (dimensionless units) as function of κa. The colloidal particle has a radius a=1000 nm and is immersed in a KCl electrolyte. The different surface electric potentials (${\hat{\Psi }}_{0}=\hat{\zeta }$) used are given in the legend. Symbols: numerical results by solving the set of electrokinetic equations. Full curves (except black one): analytical theory using Equations (75) and (76). Black curve: original analytical solution of Henry [14]. Dashed curves: analytical theory using Equations (75) and (77).

The name Henry’s function is referring to the function originally derived by Henry [14] for which Ohshima provided a simplified version [1]. Henry’s (and Oshima’s) derivation holds for low ${\hat{\Psi }}_{0}$, for which $f_{\mathrm{Henry}}$ is a function of κa only. Henry’s function $f_{\mathrm{Henry}}=\hat{\mu }/{\hat{\Psi }}_{0}$ is given in Figure 1. In the case of low potential (${\hat{\Psi }}_{0}=0.01$, giving $\Psi_{0}=$ 0.01 × 25 mV if we assume that kT/e=25 mV), one can use the expressions for the dipolar coefficient given in Appendix B to verify that, in good approximation, β=−1/2. Using this value for the dipolar coefficient and the fact that ${\hat{\Psi }}_{0}$ is low enables recovering the expression found by Ohshima for Henry’s function, as discussed in the previous section. This function (in cyan) is not to be distinguished from the black curve, which represents the original Henry’s function. It can be verified that Henry’s function (as well as the condition β=−1/2) holds for potentials up to ${\hat{\Psi }}_{0}=0.5$. For higher ${\hat{\Psi }}_{0}$, Henry’s function $f_{\mathrm{Henry}}$ becomes a function of both κa and ${\hat{\Psi }}_{0}$, as illustrated in Figure 1.

One can see that using Equation (76) for $x_{1}$ enables obtaining a better approximation at low frequencies (here, we use zero frequency) than using Equation (77), which was used in [4]. It was verified that using Equation (76) did not change the quality of the prediction at high frequencies and that the match between analytical and numerical solutions in this case is as good as in Figure 4C in [4].

The agreement between numerical calculation and analytical theory is very good for low ${\hat{\Psi }}_{0}$, even for ${\hat{\Psi }}_{0}=2.$ For higher ${\hat{\Psi }}_{0}$, the agreement for κa>10 is still good but deviates strongly from the numerical calculations at lower κa. The reason for this deviation will be illustrated in the following subsection. Note that the numerical calculations for very low κa go asymptotically to a value of 0.7 instead of 2/3=0.66, which is the Hückel limit. This deviation was also observed for the COMSOL calculations.

## 8. The Electrophoretic Mobility for Different κa

**Figure 2:**
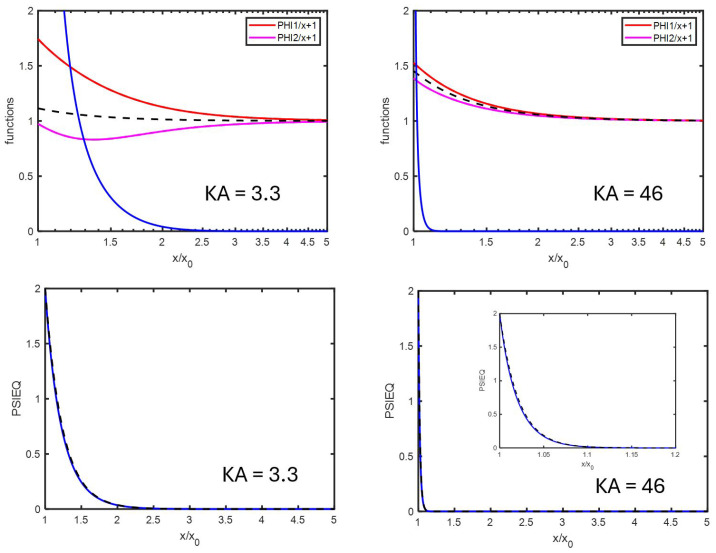
(**Upper panel**): $\left(\hat{\phi_{+}}/x+1\right)$ and $\left(\hat{\phi_{-}}/x+1\right)$ in red and magenta as function of $x/x_{0}=\kappa r/\kappa a=r/a$ for two different κa, as found by numerically solving the set of electrokinetic equations. The blue curve represents the function $\left(d{\hat{\Psi }}_{eq}/dx\right)\times \cosh\left({\hat{\Psi }}_{eq}\right)$, also evaluated numerically. The colloidal particle has a radius a=1000 nm and is immersed in a KCl electrolyte. The black dashed curve represents the function $\left(1-{\left(\kappa a\right)}^{3}\beta /x^{3}\right)$, whereby the dipolar coefficient β is the same numerically and analytically (see Figure 3). (**Lower panel**): ${\hat{\Psi }}_{eq}\left(x\right)$ for different κa as indicated in the figures: blue curves represent the numerical calculations and dashed black curves the analytical approximation Equation (38).

In order to study the behavior of the electrophoretic mobility as function of ionic strength, we concentrate on the case where ${\hat{\Psi }}_{0}=2$, for which (see Figure 1) the agreement between numerical calculation and analytical theory is very good. From Figure 2, upper panel, we can compare the functions $\left(\hat{\phi_{+}}/x+1\right)$ and $\left(\hat{\phi_{-}}/x+1\right)$ with the function $\left(1-{\left(\kappa a\right)}^{3}\beta /x^{3}\right)$ from which we deduce that Hypothesis 1 is better for higher κa. This holds for all ${\hat{\Psi }}_{0}$ tested (${\hat{\Psi }}_{0}=0.01-6$). By comparing the curves $\left(\hat{\phi_{i}}/x+1\right)$ with the curve $\left(d{\hat{\Psi }}_{eq}/dx\right)\times \cosh\left({\hat{\Psi }}_{eq}\right)$, one finds that Hypothesis 2 is fulfilled for not too low κa. This holds for all ${\hat{\Psi }}_{0}$ tested (${\hat{\Psi }}_{0}=0.01-6$). Hypothesis 3 (the fact that ${\hat{\Psi }}_{eq}$ is in good approximation given by ${\hat{\Psi }}_{eq}^{+}$) is fulfilled in all cases, as can be verified by the lower panel of Figure 2. This hypothesis breaks somewhat down for ${\hat{\Psi }}_{0}>4$, but, for such high surface potentials and especially at moderate/high κa, the decay of ${\hat{\Psi }}_{eq}$ is very fast, leading to the problem of numerical accuracy.

**Figure 3:**
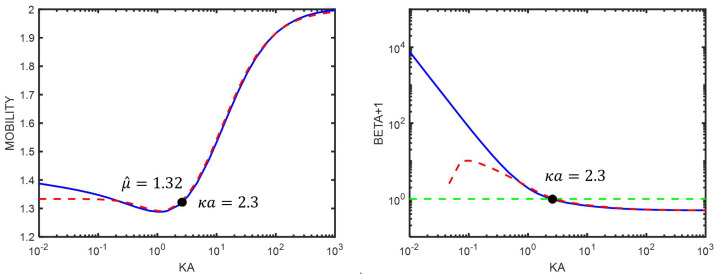
(**Left**): electrophoretic mobility $\hat{\mu }$ as function of κa. (**Right**): $\left(\beta +1\right)$ as function of κa. The blue curves are found numerically. The green dashed line is the function defined by β+1=1. The red dashed lines represent the curves according to Equations (75) and (A9). The colloidal particle has an electric surface potential ${\hat{\Psi }}_{0}=2$, a radius a=1000 nm, and is immersed in a KCl electrolyte. The black dots represent the position where β=0.

From Figure 1, we observe that, at high surface potential (also at ${\hat{\Psi }}_{0}=2$), a minimum appears in $f_{\mathrm{Henry}}$. This minimum is often reported, but its origin is worth discussing. This can be best performed by studying Equation (75). From that equation, one can directly see that the change in curvature is linked to the change in the sign of β: at low κa, we have β>0, and β is decreasing when κa is increasing. As ${\left(\kappa a\right)}^{3}/x_{1}^{3}$ is increasing, the mobility is decreasing, leading to a lowering of $\hat{\mu }$. One can estimate that, at low κa, ${\left(\kappa a\right)}^{3}/x_{1}^{3}$ scales as ${\left(\kappa a\right)}^{3}$. According to the estimation leading to Equation (96), β decreases as ${\left(\kappa a\right)}^{-2}$, which implies that $\hat{\mu }$ decreases as $\left(\kappa a\right)$. At high κa, we have β<0, and $\left|\beta \right|$ is increasing when κa is increasing, leading to an increase in $\hat{\mu }$. In Figure 3, both the mobility $\hat{\mu }$ and the dipolar coefficient β are given as a function of κa. From the points labeled in the figures, one can see that the position where β=0 is close to the position where the mobility $\hat{\mu }$ changes its curvature. Also note how high the dipolar coefficient β becomes at low κa.

### 8.1. The Role of the Double Layer

As mentioned in the previous section, the behavior of the mobility $\hat{\mu }$ can be directly linked to the dipole coefficient β. Far from the particle and its double layer, the system (particle + double layer) can be seen as an electric dipole, creating a local electric field that influences the particle’s velocity (mobility). Because the particle moves with a constant velocity, the sum of all forces exerted on the particle must be zero. This is discussed in detail in [3], where the link with the notations of Overbeek and Wiersema [15,16] is made. We adopt a different approach here. In particular, we do not restrict ourselves to low surface potentials. The colloidal particle is subjected to two forces: one electric ($F_{e}$) and one hydrodynamic ($F_{drag}$). These forces are defined by

(79) $$\begin{array}{ccc} F_{e} & \equiv & Q_{dyn}E_{0}\\ F_{drag} & = & -6\pi \eta aU \end{array}$$

where $E_{0}$ is the applied electric field, which is the electric field far away from the particle as the particle is assumed to be alone in the electrolyte solution. This electric field is different from the electric field found close to the colloidal sphere of charge *Q*. The dynamic charge $Q_{dyn}$ is defined by the relation given above. The force $F_{drag}$ is the Stokes drag force and does not account for all the hydrodynamic forces defined when studying the electrokinetic behavior of electrolytes [3]. Any “relaxation force”, due to the applied electric field or to the velocity of the particle, is accounted for in the force $F_{e}$. By using the balance of forces ($∥F_{e}∥=∥F_{drag}∥$), one obtains, using Equation (78),

(80) $$Q_{dyn}=6\pi \eta a\mu =6\pi a\varepsilon_{0}\varepsilon_{1}f_{\mathrm{Henry}}\Psi_{0}$$

The total electric field is defined, for any position around the particle, by

(81) $$\begin{array}{ccc} {\hat{E}}_{r} & = & -\left[\frac{d{\hat{\Psi }}_{eq}}{dx}\frac{kT\kappa }{eE_{0}}+\left(\frac{d\hat{\psi }\left(x\right)}{dx}-1\right)\cos\left(\theta \right)\right]\\ {\hat{E}}_{\theta } & = & \left(\frac{\hat{\psi }\left(x\right)}{x}-1\right)\sin\left(\theta \right) \end{array}$$

The velocities are given by

(82) $$\begin{array}{ccc} {\hat{u}}_{r} & = & \frac{-2}{x}\hat{h}\\ {\hat{u}}_{\theta } & = & \frac{1}{x}\frac{d}{dx}\left[x\hat{h}\right] \end{array}$$

#### 8.1.1. Hückel’s Approximation (κa≪1)

When the double layer is thick ($a\ll \kappa^{-1}$), the equilibrium electric potential is given in good approximation by

(83) $$\Psi_{eq}\left(r\right)=\Psi_{0}\frac{a}{r}$$

In this case, the potential decays over distances comparable to the particle size *a* instead of the double layer thickness $\kappa^{-1}$. This potential corresponds to the Coulomb potential around a sphere as if there were no electric double layer. In that case, one has

(84) $$Q_{dyn}=Q\;\mathrm{for}\;\kappa a\ll 1$$

Using Gauss’ relation, Equation (31), one finds

(85) $$\Psi_{0}=\frac{\sigma_{s}a}{\varepsilon_{0}\varepsilon_{1}}$$

Using the relation between charge and particle surface charge, viz

(86) $$\sigma_{s}=\frac{Q}{4\pi a^{2}}$$

one obtains

(87) $$\Psi_{0}=\frac{Q}{4\pi a\varepsilon_{0}\varepsilon_{1}a}$$

From Equation (80), one obtains

(88) $$f_{\mathrm{Henry}}=2/3$$

which implies that

(89) $$\begin{array}{ccc} \mu & = & \frac{2}{3}\frac{\varepsilon_{0}\varepsilon_{1}}{\eta }\Psi_{0}\\ & = & \frac{2}{3}\frac{\sigma_{s}a}{\eta } \end{array}$$

For colloidal particles, contrary to ions, it is quite uncommon to have small κa as a simple estimation of the double layer thickness for usual ionic strengths shows. For the purpose of illustration, the following examples are conducted with a 1000 nm colloidal particle at unrealistically low κa.

**Figure 4:**
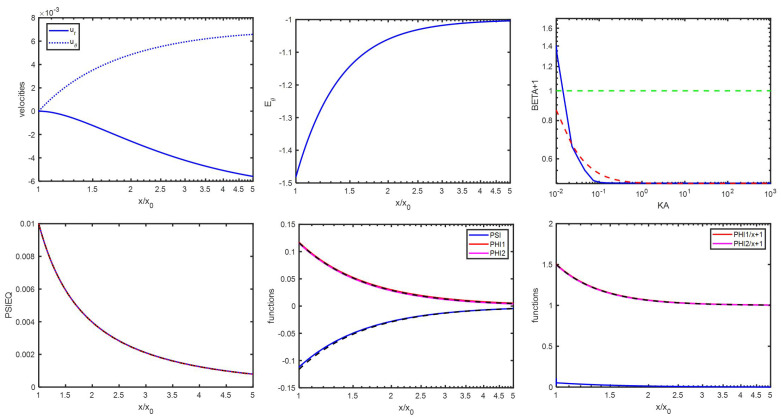
(**Top row**): velocities ${\hat{u}}_{r}$ and ${\hat{u}}_{\theta }$ as function of $x/x_{0}=r/a,$ electric field $\delta {\hat{E}}_{\theta }\left(={\hat{E}}_{\theta }\right)$, and $\left(\beta +1\right)$ as function of κa. The green dashed line is the function defined by β+1=1. The red dashed lines represent the curves according to Equations (75) and (A9). (**Bottom row**): ${\hat{\Psi }}_{eq}$ (red) $\hat{\phi_{+}}$ (red), $\hat{\phi_{-}}$ (magenta), and $\hat{\psi }$ (blue) as function of r/a, as found numerically. Dashed and dotted black curves are approximations (see text for details). The numerical functions $\left(\hat{\phi_{+}}/x+1\right)$ and $\left(\hat{\phi_{-}}/x+1\right)$ in red and magenta as function of $x/x_{0}=r/a$ can be observed to decay over the same length as the blue curve, which represents the function $\left(d{\hat{\Psi }}_{eq}/dx\right)\times \cosh\left({\hat{\Psi }}_{eq}\right)$ (also evaluated numerically), which contradicts Hypothesis 2 (Hypotheses 1 and 3 are fulfilled). The colloidal particle has an electric surface potential ${\hat{\Psi }}_{0}=0.01,$ has a radius a=1000 nm, and is immersed in a KCl electrolyte with κa=0.23.

One case of such small κa is illustrated in Figure 4, where a potential of ${\hat{\Psi }}_{0}=0.01$ is used. The curve plotted using Equation (83) is not to be distinguished from the numerical one. In Figure 4, the velocities ${\hat{u}}_{r}$ and ${\hat{u}}_{\theta }$ are plotted. Their values at long distances are ${\hat{u}}_{r}\left(x\to \infty \right)=-{\hat{u}}_{\theta }\left(x\to \infty \right)=\hat{\mu }=0.0068=2/3\times {\hat{\Psi }}_{0}$ (yielding ${\hat{\Psi }}_{0}=0.0102$ instead of ${\hat{\Psi }}_{0}=0.01)$. The same value of $\hat{\mu }=0.0068$ was obtained by evaluating the mobility using Equation (67). It can also be seen that, for the whole *x* range

(90) $$\hat{\psi }\left(x\right)=-{\hat{\phi }}_{+}\left(x\right)=-{\hat{\phi }}_{-}\left(x\right)=\frac{{\left(\kappa a\right)}^{3}\beta }{x^{2}}\;\mathrm{with}\;\beta =-1/2$$

implying that ${\hat{\psi }}^{+}=\hat{\psi }$ and ${\hat{\phi }}_{i}^{+}={\hat{\phi }}_{i}$ (note the small mismatch between analytical and numerical results at low *x* for $\hat{\psi }$). The tangential electric field ${\hat{E}}_{\theta }$ is also plotted for $\sin\left(\theta \right)=1$. It can be evaluated that

(91) $${\hat{E}}_{\theta }\left(\kappa a\right)=\left(\beta -1\right)\sin\left(\theta \right)=\frac{-3}{2}\sin\left(\theta \right)$$

which can be verified from the figure. One can also demonstrate that, for the whole *x* range

(92) $$\begin{array}{ccc} \delta {\hat{E}}_{r} & = & \left(1+\frac{{\left(\kappa a\right)}^{3}}{x^{3}}\right)\cos\left(\theta \right)\\ \delta {\hat{E}}_{\theta } & = & \left(-1-\frac{{\left(\kappa a\right)}^{3}}{2x^{3}}\right)\sin\left(\theta \right) \end{array}$$

One also obtains, from Equation (36),

(93) $$E_{2}=\frac{-3}{2}E_{0}$$

For large surface potentials and/or extremely low κa (for κa<1, see Figure 2), it was observed that the dipolar coefficient would deviate from β=−1/2 and rapidly increase with decreasing κa, becoming positive and reaching extremely high values. In that case as well, as indicated in Figure 2, Hypothesis 2 does not hold anymore. Despite the very high values of β, as β scales roughly with ${\left(\kappa a\right)}^{-2}$ and the prefactor in Equation (75) with ${\left(\kappa a\right)}^{3},$ Equation (89) remains satisfied. A rough estimation to obtain the dependence of β on $\kappa^{-2}$ can be achieved as follows. The characteristic timescale associated with the double layer is

(94) $$\tau ∼\frac{1}{D\kappa^{2}}$$

where *D* is an ionic diffusion coefficient. The velocity associated with the deformation of the double layer (for small κa) is

(95) $$v∼\frac{Q}{\eta a}E_{0}$$

This implies that the dipolar coefficient can be estimated by

(96) $$\beta ∼P∼Q\frac{v}{\tau }$$

indeed yielding $\beta ∼\kappa^{-2}.$

#### 8.1.2. Smoluchowski’ s Approximation (κa≫1)

When the double layer is thin ($a\gg \kappa^{-1}$), we can approximate that the double layer is not deformed under the influence of the applied electric field. The ionic densities will predominantly vary in the θ direction. When the equilibrium potential is low, it can be given by

(97) $$\Psi_{eq}\left(r\right)≃\Psi_{0}\exp\left(-\kappa \left(r-a\right)\right)$$

as the characteristic distance over which the electric field is non-zero is $\left(r-a\right)≃\kappa^{-1}$, which implies that

(98) $$\frac{a}{r}=\frac{a}{a+\left(r-a\right)}≃\frac{\kappa a}{\kappa a+1}≃1$$

Equation (97) is also the distribution found in the case of a planar surface. The main difference with the planar case, as we will see, is that the electrophoretic mobility is mainly influenced by the asymmetric ionic distribution in the θ direction due to the application of an electric field (in the planar case, because of symmetry, there cannot be ionic gradients in the direction parallel to the plane). From Gauss’ relation given by Equation (31), it then follows that

(99) $$\Psi_{0}=\frac{\sigma_{s}}{\varepsilon_{0}\varepsilon_{1}\kappa }$$

We now do not make the assumption that the potential is low. One can consider the system composed of the charged colloidal particle and its extremely thin double layer as an electroneutral system, for which the electric potential δΨ should obey the Laplace equation and the associated boundary condition (where $a^{+}=a+\kappa^{-1}$)

(100) $$\begin{array}{ccc} \nabla^{2}\delta \Psi \left(r\right) & = & 0\\ {\left(\nabla_{r}\delta \Psi \right)}_{r=a^{+}} & = & 0 \end{array}$$

Solving these two equations yields

(101) $$\begin{array}{ccc} \delta \Psi^{+} & = & -\left(1+\frac{1}{2}\frac{a^{3}}{r^{3}}\right)E_{0}\cdot r\\ E_{\theta }^{+} & = & {\left(\frac{-1}{r}\frac{\partial \delta \Psi }{\partial \theta }\right)}_{r=a^{+}}=\frac{-3}{2}E_{0}\sin\left(\theta \right) \end{array}$$

As the pressure does not vary in the tangential (θ) direction, using the fact that $\nabla \Psi =-E_{\theta }^{+}e_{\theta }$, and using Poisson ’s equation in Equation (22), the Navier–Stokes equation, Equation (51), becomes in the tangential direction

(102) $$\eta \frac{\partial^{2}u_{\theta }^{+}}{\partial r^{2}}=\varepsilon_{0}\varepsilon_{1}\frac{\partial^{2}\Psi }{\partial r^{2}}E_{\theta }^{+}$$

Integrating Equation (102) between an arbitrary position *r* and r→∞, using the fact that $\partial u_{\theta }^{+}/\partial r\left(r\to \infty \right)=0$ and $\partial \Psi /\partial r\left(r\to \infty \right)=0$ yields

(103) $$\eta \frac{\partial u_{\theta }^{+}}{\partial r}=\varepsilon_{0}\varepsilon_{1}\frac{\partial \Psi }{\partial r}E_{\theta }^{+}$$

**Figure 5:**
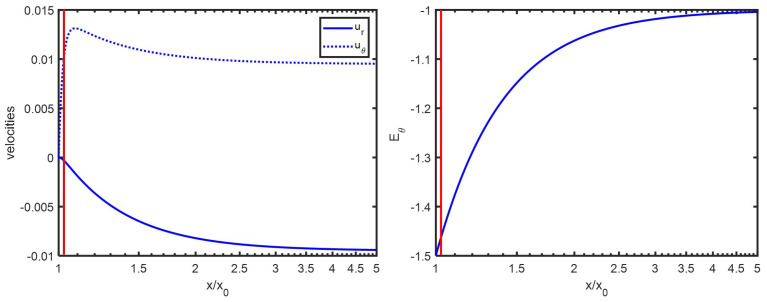
Velocities ${\hat{u}}_{r}$ and ${\hat{u}}_{\theta }$ and electric field $\delta {\hat{E}}_{\theta }\left(={\hat{E}}_{\theta }\right)$ as function of $x/x_{0}=r/a.$ The red line indicates the position where $x=\kappa a^{+}=\kappa a+1$. The approximation made in the derivation of the Smoluchowski expression, i.e., $E_{\theta }=E_{\theta }^{+}$, seems to be justified. The colloidal particle has an electric surface potential ${\hat{\Psi }}_{0}=0.01,$ has a radius a=1000 nm, and is immersed in a KCl electrolyte with κa=46.

Integrating again, this time between r=a and $r=a^{+}$, making the assumption that $E_{\theta }=E_{\theta }^{+}$ in the double layer (see Figure 5), we find

(104) $$\eta \left[0-u_{\theta }^{+}\left(a^{+}\right)\right]=\varepsilon_{0}\varepsilon_{1}\left[\Psi \left(a\right)-0\right]E_{\theta }^{+}$$

The tangential fluid velocity $u_{\theta }^{+}\left(a^{+}\right)$ can be estimated by realizing that, since there is no net force on the liquid and no pressure gradient is applied, the liquid flow must have a potential nature

(105) $$u\left(r\right)=-\nabla \Phi \left(r\right)$$

As the fluid is incompressible, ∇·u=0 and

(106) $$\nabla^{2}\Phi \left(r\right)=0$$

As the liquid cannot penetrate the colloidal particle,

(107) $${\left(\nabla_{r}\Phi \right)}_{r=a}=0$$

Combining these equations yields

(108) $$\begin{array}{ccc} \Phi & = & -\left(1+\frac{1}{2}\frac{a^{3}}{r^{3}}\right)u_{\infty }\cdot r\\ u_{\theta }^{+} & = & {\left(\frac{-1}{r}\frac{\partial \Phi }{\partial \theta }\right)}_{r=a^{+}}=\frac{-3}{2}u_{\infty }\sin\left(\theta \right)=\frac{3}{2}\mu E_{0}\sin\left(\theta \right) \end{array}$$

From Equations (101), (104) and (108), we obtain

(109) $$-\eta \frac{3}{2}\mu E_{0}\sin\left(\theta \right)=\varepsilon_{0}\varepsilon_{1}\Psi_{0}\frac{-3}{2}E_{0}\sin\left(\theta \right)$$

yielding

(110) $$\mu =\frac{\varepsilon_{0}\varepsilon_{1}}{\eta }\Psi_{0}$$

from which we deduce that

(111) $$f_{\mathrm{Henry}}=1$$

A thorough discussion about the Smoluchowski limit is given in [9].

From Equation (80), one obtains

(112) $$Q_{dyn}=6\pi a\varepsilon_{0}\varepsilon_{1}\Psi_{0}$$

In order to obtain a relation between $\Psi_{0}$ and *Q*, one uses Equation (A15) found in Appendix B, which yields

(113) $$Q=\frac{4\pi \varepsilon_{0}\varepsilon_{1}kTa}{e}\kappa a\left[2\sinh\left(\frac{{\hat{\Psi }}_{0}}{2}\right)+\frac{4}{\kappa a}\tanh\left(\frac{{\hat{\Psi }}_{0}}{4}\right)-\frac{{\hat{\Psi }}_{0}}{\kappa a}\right]$$

This relation provides a good estimate for κa≥0.5. In the limit of low ${\hat{\Psi }}_{0}$, one obtains

(114) $$Q=4\pi \varepsilon_{0}\varepsilon_{1}a\kappa a\Psi_{0}$$

from which it is deduced that

(115) $$Q_{dyn}=\frac{3}{2}\frac{Q}{\kappa a}\;\;\;\mathrm{for}\;\;\;{\hat{\Psi }}_{0}\ll 1\;\mathrm{and}\;\kappa a\gg 1$$

## 9. Particle with a Constant Surface Charge

Many articles present theoretical results for the electrophoretic motion of a colloidal particle with a constant surface potential as function of κa. This is done for convenience as a constant surface potential implies a Dirichlet boundary condition (Equation (2)), whereas a constant surface charge implies using a Neumann boundary condition (Equation (31)), which is a bit more complicated to implement. Nonetheless, in practice, colloidal particles tend to have a relatively constant surface charge as a function of ionic strength (for a given pH) and therefore represent an interesting case to study [6,17,18].

**Figure 6:**
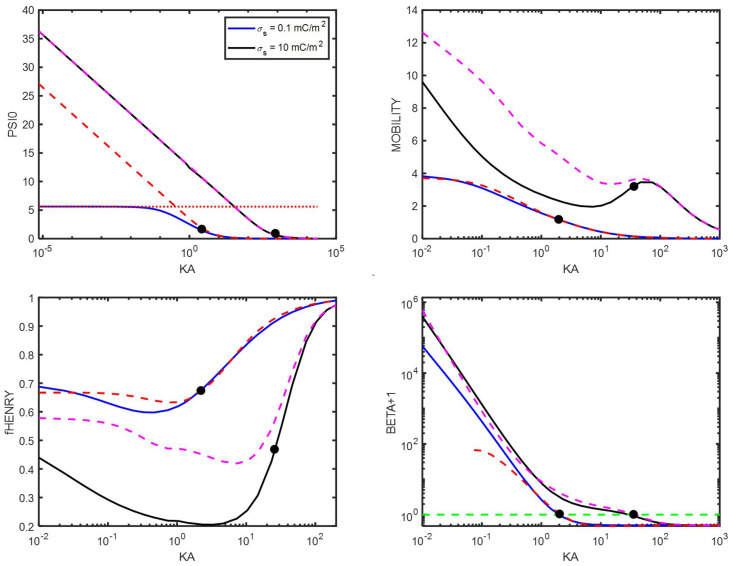
The black and blue curves are found numerically. (**Top row**): surface electric potential ${\hat{\Psi }}_{0}$ and electrophoretic mobility $\hat{\mu }$ as function of κa. The dotted and dashed lines for ${\hat{\Psi }}_{0}$ represent approximations (see text for details). (**Bottom row**): Henry’s function $f_{\mathrm{Henry}}$ and $\left(\beta +1\right)$ as function of κa. The green dashed line is the function defined by β+1=1. The red and magenta dashed curves for $\hat{\mu },\;f_{\mathrm{Henry}}$, and β represent the curves according to Equations (75) and (A9). The colloidal particles have a radius a=1000 nm and are immersed in a KCl electrolyte. The black dots represent the position where β=0.

In Figure 6, results are shown for a particle of constant surface charge.

One notes that the curve for Henry’s function $f_{\mathrm{Henry}}$ is very similar to the one given for the constant potential case (see Figure 1) for the same reason that, in the limit of low and high κa, the function reaches Hückel and Smoluchowski limits (which are independent of the fact that one considers a constant surface potential or a constant surface charge).

The approximated dashed functions for high ${\hat{\Psi }}_{0}$ are found using the following relation (see a more accurate formulation, i.e., Equation (A15), in Appendix B)

(116) $$q^{*}=\frac{e\sigma_{s}}{\varepsilon_{0}\varepsilon_{1}kT\kappa }≃2\sinh\left(\frac{{\hat{\Psi }}_{0}}{2}\right)$$

Inverting this equation provides the desired approximation for ${\hat{\Psi }}_{0}$ as function of $\sigma_{s}$ and κ. For low surface charge ($\sigma_{s}=0.1$ mC/m^2^), the surface potential reaches a constant value below κa=1. This can be understood by estimating the potential/charge relation for low ${\hat{\Psi }}_{0}$ by using Equations (31) and (38):

(117) $${\hat{\Psi }}_{0}\left(\frac{1}{a}+\kappa \right)=\frac{e\sigma_{s}}{\varepsilon_{0}\varepsilon_{1}kT}$$

At low surface charge (implying low surface potential) and low κa (implying no effect of the double layer on the surface potential), the surface potential ${\hat{\Psi }}_{0}$ can be approximated by

(118) $${\hat{\Psi }}_{0}≃\frac{e\sigma_{s}a}{\varepsilon_{0}\varepsilon_{1}kT}$$

For $\sigma_{s}=0.1$ mC/m^2^, this yields ${\hat{\Psi }}_{0}≃5.6$, which corresponds to the value found numerically and represented by the red dotted line.

For a particle with a constant surface charge, at low κa, the electrophoretic mobility is given by, using Equations (89) and (117),

(119) $$\mu_{\mathrm{low}\;\kappa a}=\frac{2}{3}\frac{\sigma_{s}a}{\eta }$$

This equation is valid for κa values that correspond to physically unrealistic ionic strengths for colloidal particles. In the examples chosen, for the low surface charges of −0.1 mC/m^2^ and −0.2 mC/m^2^, the relation is valid below a concentration of 10^−8^ mM (!). The limit is not reached for the higher surface charges, not even at 10^−15^ mM. In that case, the curves are superposed below κa=1. At high and increasing κa, the electrophoretic mobility of a particle with constant surface charge is decreasing until reaching zero as its surface electric potential ${\hat{\Psi }}_{0}$ is decreasing rapidly with ionic strength (see Figure 4). For low surface potentials, the decrease is modeled by using Equations (110) and (117)

(120) $$\mu_{\mathrm{high}\;\kappa a}=\frac{\sigma_{s}}{\eta \kappa }$$

**Figure 7:**
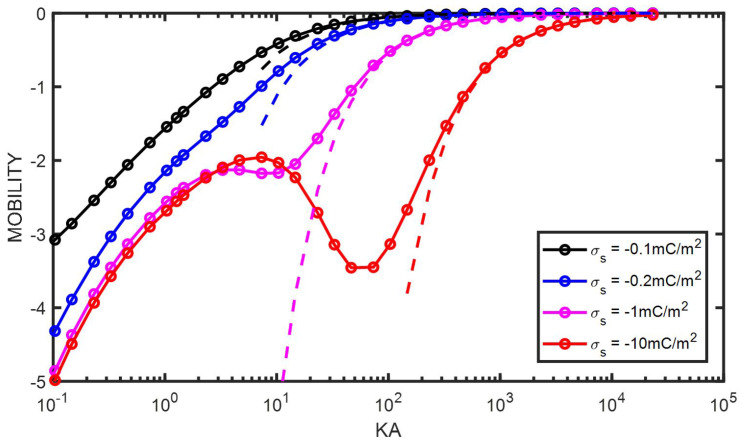
Electrophoretic mobility $\hat{\mu }$ found numerically as function of κa for different values of the (negative) surface charge $\sigma_{s}$ as given in the legend. The dashed lines are plotted according to Equation (120). The colloidal particle has a radius a=1000 nm and is immersed in a KCl electrolyte.

In Figure 7, it is shown that Equation (120) indeed enables approximating the electrophoretic mobility of colloidal particles for any (constant) surface charge at high κa. Fitting the electrophoretic mobility data for charged colloidal particles with a constant surface charge at high ionic strength with Equation (120) therefore enables obtaining the surface charge without the need for numerical calculations.

## 10. Conclusions

In the present article, a new analytical equation has been presented for the electrophoretic mobility of a colloidal sphere. It has been shown that the equation predicts the electrophoretic mobility well for the whole range of κa provided that the electric surface potential (where the slip layer is defined) is less than 50 mV (${\hat{\Psi }}_{0}=2$). For higher surface potentials, the analytical prediction deviates from the numerical results below κa=10, and the deviations increase with increasing surface potentials. For experimental conditions, where usually κa>10, the proposed equation is an easy-to-implement alternative to the full numerical solution. From the study of the analytical equation, it was shown that the fact that the electrophoretic mobility decreases with increasing κa for low κa and subsequently increases with increasing κa for high κa is linked to the change in sign of the dipolar coefficient. In the region of intermediate κa, the electrophoretic mobility experiences a change in curvature associated with the presence of a maximum (or minimum, depending on the charge of the particle). As many suspensions consist of colloidal particles with a relatively constant surface charge (for a given pH), it was shown that a simple equation (Equation (120)) enables estimating this surface charge by fitting the electrophoretic mobility as a function of ionic strength at high ionic strength (for κa>10 in Figure 7).

## Figures and Tables

**Figure A1:**
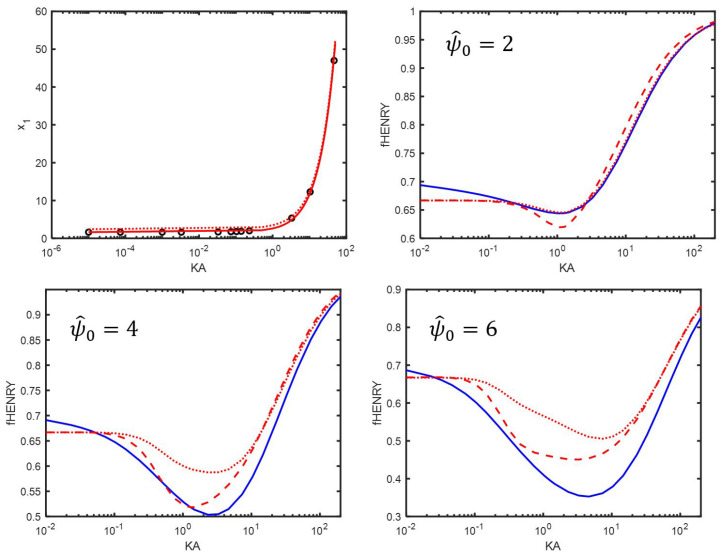
(**Top row, left figure**): length $x_{1}$ defined as the position where $F\left(x\right)$ defined by Equation (A17) is optimum. The dotted red curve represents Equation (76) and the full red curve represents Equation (A18). (**Other figures**): Henry’s functions $f_{\mathrm{Henry}}$ for different surface potentials ${\hat{\Psi }}_{0}$, as indicated in each figure. Blue curves: numerical results. Dotted red curve: results using Equations (75) and (76). Dashed red curves: results using Equations (75) and (A18).

**Figure A2:**
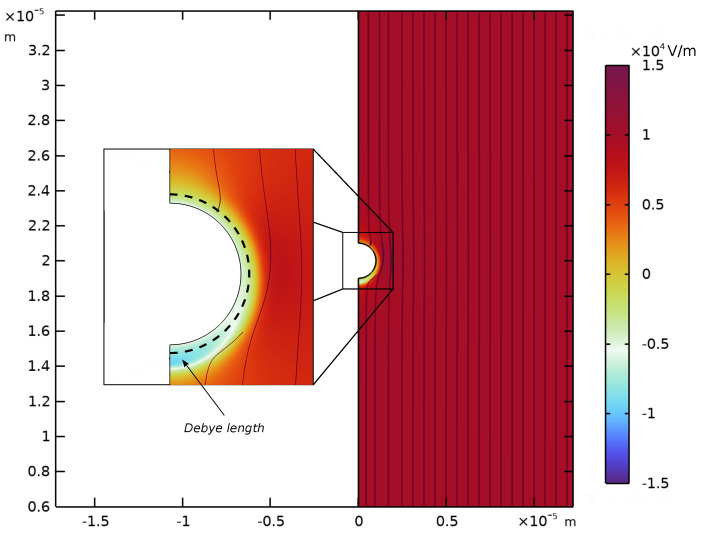
Difference between the magnitude of the electric field resulting from the applied electric field ($∥\delta E∥$ as described in Equation (6)) and the magnitude of the electric field in the absence of applied electric field ($∥\nabla \Psi_{eq}∥$). The magnitude of the applied electric field is $E_{0}$ = 10,000 V/m for a particle radius a=1000 nm, a particle surface charge of 0.1 mC/m^2^, and κa = 10.39. The streamlines in the figure represent the total electric field.

**Figure A3:**
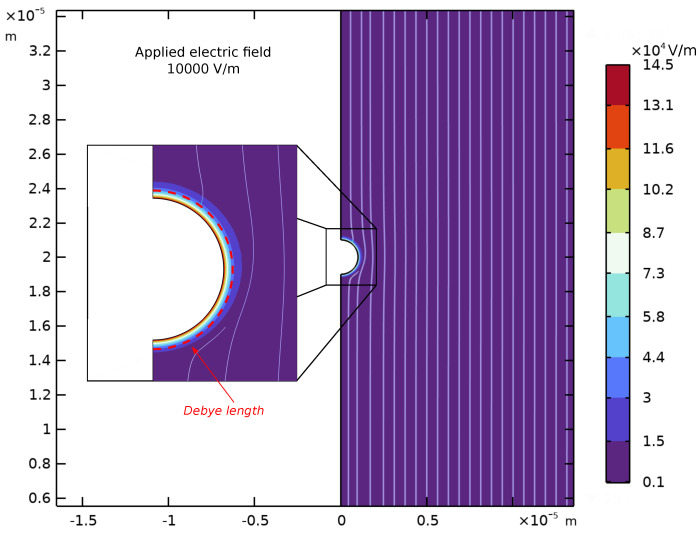
Magnitude and streamlines of the total electric field ($∥E∥$) resulting from the application of $E_{0}=E_{0}e_{z}$ with $E_{0}$ = 10,000 V/m for a particle radius a=1000 nm, a particle surface charge of 0.1 mC/m^2^, and κa = 10.39.

**Figure A4:**
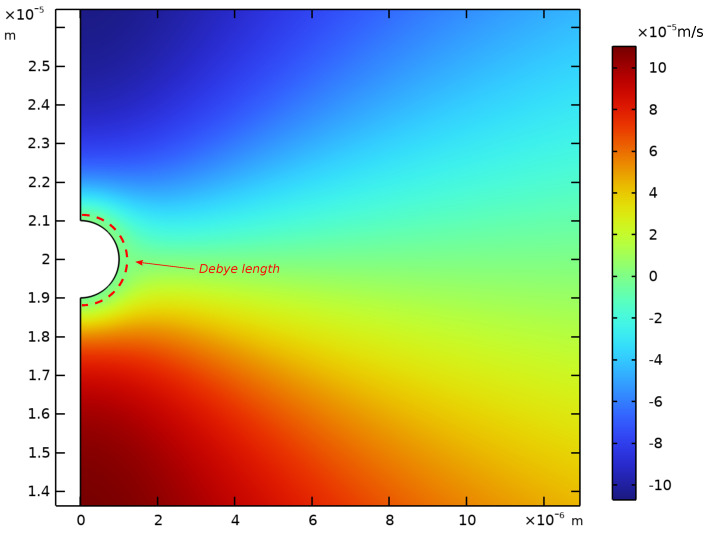
Magnitude of the r-component of the velocity, $u_{r}$, resulting from the application of $E_{0}=E_{0}e_{z}$ with $E_{0}$ = 10,000 V/m for a particle radius a=1000 nm, a particle surface charge of 0.1 mC/m^2^, and κa = 10.39.

**Figure A5:**
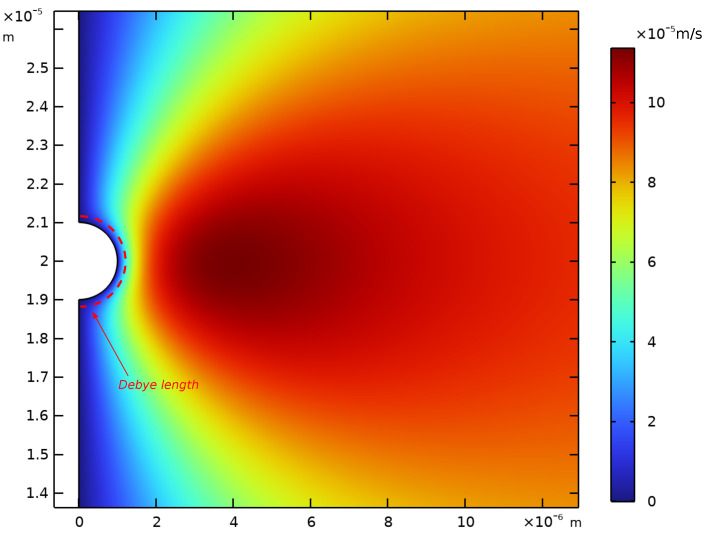
Magnitude of the θ-component of the velocity, $u_{\theta }$, resulting from the application of $E_{0}=E_{0}e_{z}$ with $E_{0}$ = 10,000 V/m for a particle radius a=1000 nm, a particle surface charge of 0.1 mC/m^2^, and κa = 10.39.

**Figure A6:**
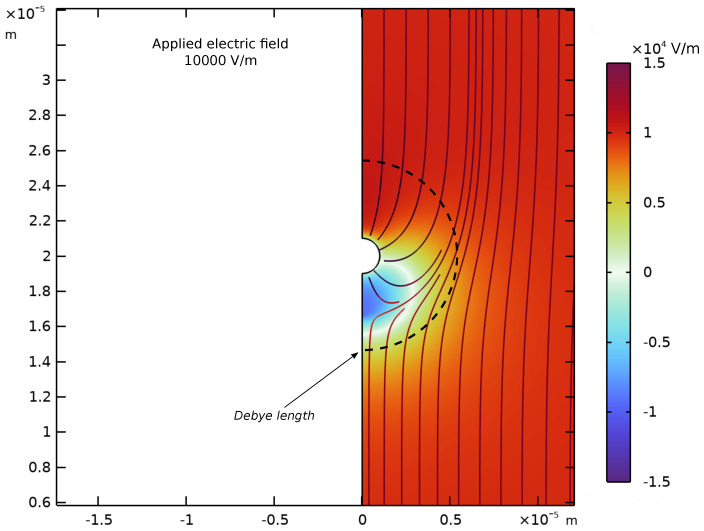
Difference between the magnitude of the electric field resulting from the applied electric field ($∥\delta E∥$ as described in Equation (6)) and the magnitude of the electric field in the absence of applied electric field ($∥\nabla \Psi_{eq}∥$). The magnitude of the applied electric field is $E_{0}$ = 10,000 V/m for a particle radius a=1000 nm, a particle surface charge of 0.1 mC/m^2^, and κa = 0.23. The streamlines in the figure represent the total electric field **E**.

**Figure A7:**
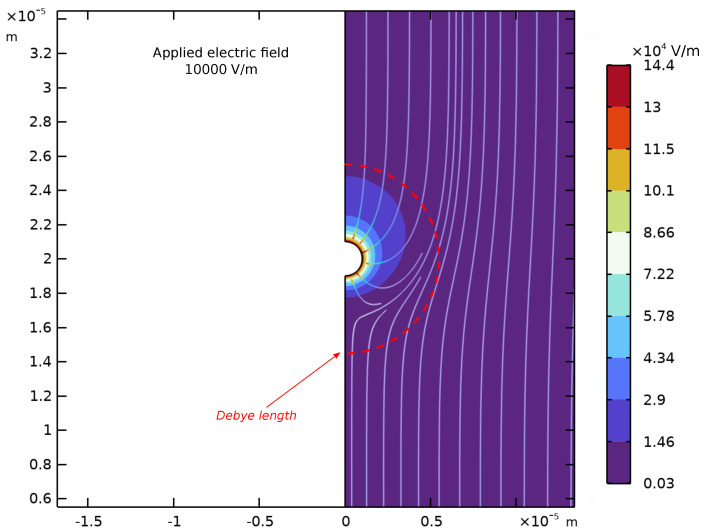
Magnitude and streamlines of the total electric field ($∥E∥$) resulting from the application of $E_{0}=E_{0}e_{z}$ with $E_{0}$ = 10,000 V/m for a particle radius a=1000 nm, a particle surface charge of 0.1 mC/m^2^, and κa = 0.23.

**Figure A8:**
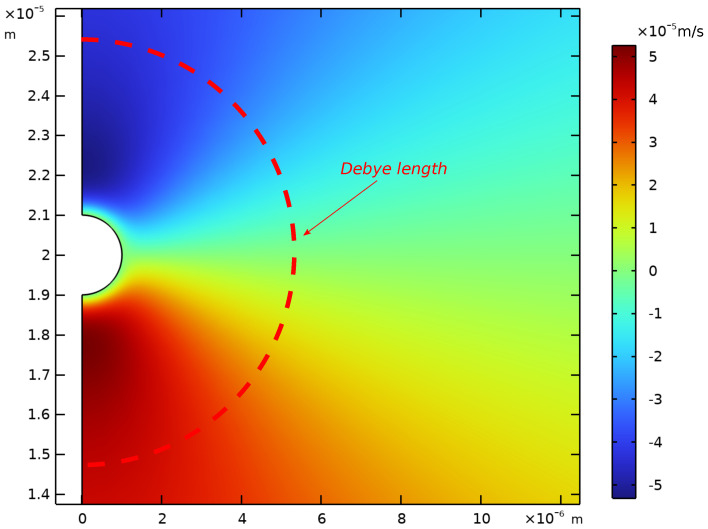
Magnitude of the r-component of the velocity, $u_{r}$, resulting from the application of $E_{0}=E_{0}e_{z}$ with $E_{0}$ = 10,000 V/m for a particle radius a=1000 nm, a particle surface charge of 0.1 mC/m^2^, and κa = 0.23.

**Figure A9:**
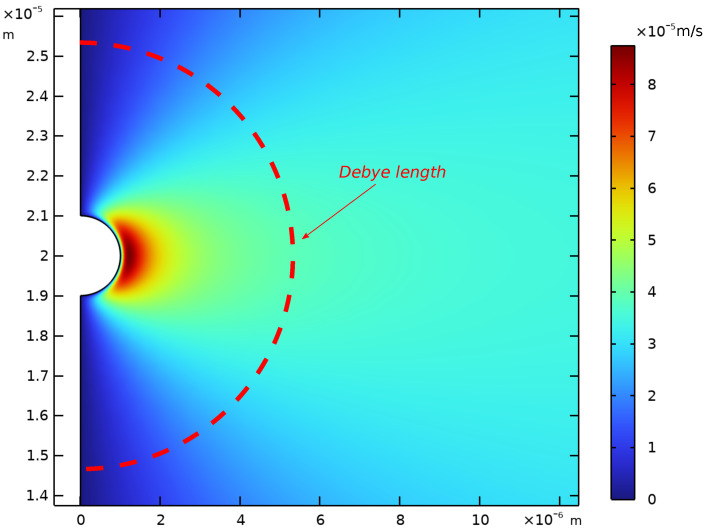
Magnitude of the θ-component of the velocity, $u_{\theta }$, resulting from the application of $E_{0}=E_{0}e_{z}$ with $E_{0}$ = 10,000 V/m for a particle radius a=1000 nm, a particle surface charge of 0.1 mC/m^2^, and κa = 0.23.

## Data Availability

Data is contained within the article.

## Appendix A. Equivalence with Previous Work

For comparison with the analytical results presented in [4,8], the following variables are introduced:

(A1) $$\begin{array}{ccc} n_{n} & = & n_{+}+n_{-}\;(\mathrm{similarly}:\;n_{n,eq}=n_{+,eq}+n_{-,eq})\\ n_{c} & = & n_{+}+\left(\frac{z_{-}}{z_{+}}\right)n_{-}\;(\mathrm{similarly}:\;n_{c,eq}=n_{+,eq}+\left(\frac{z_{-}}{z_{+}}\right)n_{-,eq}) \end{array}$$

We can make the equivalence

$$\begin{array}{ccc} n_{n,eq} & = & \nu_{+}n_{\infty }\exp\left(-z_{+}{\hat{\Psi }}_{eq}\right)+\nu_{-}n_{\infty }\exp\left(-z_{-}{\hat{\Psi }}_{eq}\right)\\ & = & 2n_{\infty }\cosh\left({\hat{\Psi }}_{eq}\right)\;\mathrm{for}\;a\;1–1\;\mathrm{electrolyte}\\ n_{c,eq} & = & \nu_{+}n_{\infty }\left[\exp\left(-z_{+}{\hat{\Psi }}_{eq}\right)-\exp\left(-z_{-}{\hat{\Psi }}_{eq}\right)\right]\\ & = & -2n_{\infty }\sinh\left({\hat{\Psi }}_{eq}\right)\;\mathrm{for}\;a\;1–1\;\mathrm{electrolyte} \end{array}$$

Similarly,

(A2) $$\begin{array}{ccc} n_{n} & = & \frac{-e}{kT\kappa }\left[z_{+}n_{+,eq}\left(\hat{\psi }+\hat{\phi_{+}}\right)+z_{-}n_{-,eq}\left(\hat{\psi }+\hat{\phi_{-}}\right)\right]\\ n_{c} & = & \frac{-e}{kT\kappa }\left[z_{+}n_{+,eq}\left(\hat{\psi }+\hat{\phi_{+}}\right)+\left(\frac{z_{-}}{z_{+}}\right)z_{-}n_{-,eq}\left(\hat{\psi }+\hat{\phi_{-}}\right)\right] \end{array}$$

For a 1–1 electrolyte, these equations reduce to

(A3) $$\begin{array}{ccc} n_{n} & = & \frac{\varepsilon_{0}\varepsilon_{1}}{e}\kappa \left[\sinh\left({\hat{\Psi }}_{eq}\right)\hat{\psi }-\frac{1}{2}\left(\exp\left(-{\hat{\Psi }}_{eq}\right)\hat{\phi_{+}}-\exp\left({\hat{\Psi }}_{eq}\right)\hat{\phi_{-}}\right)\right]\\ n_{c} & = & \frac{\varepsilon_{0}\varepsilon_{1}}{e}\kappa \left[-\cosh\left({\hat{\Psi }}_{eq}\right)\hat{\psi }-\frac{1}{2}\left(\exp\left(-{\hat{\Psi }}_{eq}\right)\hat{\phi_{+}}+\exp\left({\hat{\Psi }}_{eq}\right)\hat{\phi_{-}}\right)\right] \end{array}$$

It can be shown that, far from the particle [8],

$$L\psi =\frac{1}{r}\frac{d^{2}}{dr^{2}}\left(r\psi \right)-\frac{2\psi }{r^{2}}=\frac{-e}{\varepsilon_{0}\varepsilon_{1}}\sum z_{i}n_{i}=\frac{-ez_{+}}{\varepsilon_{0}\varepsilon_{1}}n_{c}$$

with

(A4) $$\begin{array}{ccc} n_{n}^{+}\left(r\right) & = & \frac{C_{n}}{r^{2}}\\ n_{c}^{+}\left(r\right) & = & C_{c}\frac{1+\kappa r}{r^{2}}\exp\left(-\kappa (r-a)\right)≃0\;\mathrm{beyond}\;\mathrm{the}\;\mathrm{double}\;\mathrm{layer} \end{array}$$

yielding

(A5) $$L\psi^{+}=0$$

The solution of this equation is

(A6) $$\begin{array}{ccc} \psi^{+}\left(r\right) & = & \frac{a^{3}\beta }{r^{2}}\\ {\hat{\psi }}^{+}\left(x\right) & = & \frac{{\left(\kappa a\right)}^{3}\beta }{x^{2}} \end{array}$$

where β is the dipolar coefficient. As

(A7) $$n_{c}^{+}=\frac{-ez_{+}\nu_{+}n_{\infty }}{kT\kappa }\left[\left(\hat{\psi^{+}}+\hat{\phi_{+}^{+}}\right)-\left(\frac{z_{-}}{z_{+}}\right)\left(\hat{\psi^{+}}+\hat{\phi_{-}^{+}}\right)\right]$$

it follows that, using Equation (16) and the fact that $n_{i}^{+}≃0$

(A8) $$\phi_{+}^{+}\left(r\right)=\phi_{-}^{+}\left(r\right)=\frac{-a^{3}\beta }{r^{2}}$$

## Appendix B. An Analytical Expression for β

An analytical expression for β has been derived in [4]. In [8], the link was made between dielectric spectroscopy experiments and dipolar coefficient β. The analytical expression for the dipolar coefficient for ω=0 for a 1–1 electrolyte is given by

(A9) $$\beta =\frac{-K_{1}+2\left[K_{//}+K_{U}\right]+K_{⊥}}{2K_{1}+2\left[K_{//}{\left(\kappa a/x_{0}\right)}^{3}+K_{U}{\left(\kappa a/x_{1}\right)}^{3}-K_{⊥}\right]}$$

The conductivities $K_{i}$ are defined by

(A10) $$\begin{array}{ccc} K_{1} & = & \varepsilon_{1}\varepsilon_{0}\kappa^{2}D_{0}\\ K_{//} & = & -K_{1}I_{n,eq}-\frac{\left[I_{c,eq}^{2}-I_{n,eq}^{2}\right]}{{\left(x_{0}/\kappa a\right)}^{3}}\\ K_{⊥} & = & \frac{K_{1}I_{n,eq}}{{\left(x_{0}/\kappa a\right)}^{3}}\\ K_{U} & = & -K_{1}m{\hat{\Psi }}_{0}I_{c,eq}\left[\frac{1}{4{\left(x_{0}/\kappa a\right)}^{3}}-1\right] \end{array}$$

where

(A11) $$\begin{array}{ccc} x_{0} & = & 1+\kappa a+\frac{3}{\kappa a}\exp\left(\frac{-{\hat{\Psi }}_{0}}{2}\right)\\ x_{1} & = & \kappa a+\frac{2.5}{1+2\exp\left(-\kappa a\right)\exp\left(-{\hat{\Psi }}_{0}\right)}\\ m & = & \frac{2}{3}\frac{\varepsilon_{0}\varepsilon_{1}{\left(kT\right)}^{2}}{\eta D_{0}e^{2}}\\ D_{0} & = & \frac{z_{+}D_{+}-z_{-}D_{-}}{z_{+}-z_{-}} \end{array}$$

and

(A12) $$\begin{array}{ccc} I_{c,eq} & = & \frac{1}{{\left(\kappa a\right)}^{2}}\int_{\kappa a}^{x_{0}}x\sinh\left(\Psi_{eq}\right)dx\\ I_{n,eq} & = & \frac{-1}{{\left(\kappa a\right)}^{2}}\int_{\kappa a}^{x_{0}}x\left[\cosh\left({\hat{\Psi }}_{eq}\right)-1\right]dx \end{array}$$

Approximated analytical expressions for β, $I_{c,eq}$, and $I_{n,eq}$ can be found in [4,8].

### Appendix B.1. Low ${\hat{\Psi }}_{0}$

In the case of low surface potentials, one finds that [8]

(A13) $$\begin{array}{ccc} I_{c,eq} & ∼ & \frac{{\hat{\Psi }}_{0}}{\kappa a}\\ I_{n,eq} & ∼ & 0 \end{array}$$

yielding

(A14) $$\begin{array}{ccc} K_{//} & = & {\left({\hat{\Psi }}_{0}\right)}^{2}\frac{\kappa a}{x_{0}^{3}}\\ K_{⊥} & = & 0\\ K_{U} & = & -K_{1}m{\left({\hat{\Psi }}_{0}\right)}^{2}\frac{1}{\kappa a}\left[\frac{1}{4{\left(x_{0}/\kappa a\right)}^{3}}-1\right] \end{array}$$

### Appendix B.2. High ${\hat{\Psi }}_{0}$

For high surface potentials, one finds that [8]

(A15) $$\begin{array}{ccc} I_{c,eq} & ∼ & \frac{q^{*}}{\kappa a}=-I_{n,eq}\\ q^{*} & = & 2\sinh\left(\frac{{\hat{\Psi }}_{0}}{2}\right)+\frac{4}{\kappa a}\tanh\left(\frac{{\hat{\Psi }}_{0}}{4}\right)-\frac{{\hat{\Psi }}_{0}}{\kappa a} \end{array}$$

where $q^{*}$ is the dimensionless surface charge density, and one finds that

(A16) $$\begin{array}{ccc} K_{//} & = & K_{1}\frac{q^{*}}{\kappa a}\\ K_{⊥} & = & \frac{-K_{1}}{{\left(x_{0}/\kappa a\right)}^{3}}\frac{q^{*}}{\kappa a}\\ K_{U} & = & -K_{1}m{\hat{\Psi }}_{0}\frac{q^{*}}{\kappa a}\left[\frac{1}{4{\left(x_{0}/\kappa a\right)}^{3}}-1\right] \end{array}$$

## Appendix C. An Estimation of *x*_1_

The length $x_{1}$ is defined as the position where the function

(A17) $$F\left(x\right)=\left(1-3\frac{x^{2}}{{\left(\kappa a\right)}^{2}}+2\frac{x^{3}}{{\left(\kappa a\right)}^{3}}\right)\frac{1}{\sum z_{i}^{2}\nu_{i}}\frac{d}{dx}\left[\sum z_{i}\nu_{i}\exp\left(-z_{i}{\hat{\Psi }}_{eq}\right)\right]$$

is optimum, see discussion under Equation (72). From the numerical results, the values for $x_{1}$ for a large range of κa and ${\hat{\Psi }}_{0}$ were tested. In Figure A1, the values for $x_{1}$ for ${\hat{\Psi }}_{0}=4$ are presented. Similar figures were obtained for ${\hat{\Psi }}_{0}=1,2,3,5$, and 6. The fit given by Equation (76) does not match the numerical result for low κa. For low κa, irrespective of ${\hat{\Psi }}_{0}$, it was found that $x_{1}=1.63.$ To better approximate the numerically found $x_{1}$, it is necessary to adapt Equation (76) as follows:

(A18) $$x_{1}=\kappa a+\frac{1.63}{1+2\exp\left(-\kappa a\right)\exp\left(-{\hat{\Psi }}_{0}/\kappa a\right)}$$

Using Equation (A18) (see Figure A1), it is possible to obtain a somewhat better fit at high ${\hat{\Psi }}_{0}$ (=4 or 6), but it worsens the fit for lower ${\hat{\Psi }}_{0}$ (=2). Therefore, it was decided to use Equation (76).

## Appendix D. Spatial Representation

Some of the spatial representations of the quantities analyzed for selected values of κa are plotted in this section. The numerical calculations were performed using the Finite Element software COMSOL Multiphysics v. 6.3 [13].
